# Immunity against a Japanese local strain of porcine reproductive and respiratory syndrome virus decreases viremia and symptoms of a highly pathogenic strain

**DOI:** 10.1186/s12917-021-02863-4

**Published:** 2021-04-13

**Authors:** Hiroshi Iseki, Kenji Kawashima, Tomoyuki Shibahara, Masaji Mase

**Affiliations:** 1 Division of Viral Disease and Epidemiology, National Institute of Animal Health, National Agriculture and Food Research Organization, 3-1-5 Kannondai, Tsukuba, Ibaraki, 305-0856 Japan; 2 Division of Pathology and Pathophysiology, National Institute of Animal Health, National Agriculture and Food Research Organization, 3-1-5 Kannondai, Tsukuba, Ibaraki, 305-0856 Japan; 3 Department of Veterinary Science, Graduate School of Life and Environmental Sciences, Osaka Prefecture University, 1-58 Rinku-oraikita, Izumisano, Osaka, 598-8531 Japan; 4 United Graduate School of Veterinary Sciences, Gifu University, 1-1 Yanagido, Gifu, Gifu 501-1193 Japan

**Keywords:** Cross-protection, Highly pathogenic, Immunity, Pathogenicity, Pig, Porcine reproductive and respiratory syndrome virus, PRRS, Vaccine

## Abstract

**Background:**

The type 2 highly pathogenic porcine reproductive and respiratory syndrome virus (HP-PRRSV) has spread throughout countries of southeast Asia, where it has caused severe economic losses. Even countries presently free of PRRSV are at high risk for infection and spread of this virus. Some of these countries, including Japan, have broad epidemics of the local type 2 PRRSV, creating chronic pathogenicity in the domestic pig population. The present study aimed to evaluate the protective efficacy of immunity by infection with a Japanese field isolate, EDRD1, against heterologous challenge with a Vietnamese HP-PRRSV field strain. To this end, four groups of PRRSV-negative crossbreed piglets were used for a challenge study. Groups 1 and 2 were inoculated with EDRD1 via the intranasal route. After 26 days, Groups 2 and 3 were inoculated with HP-PRRSV via the same route. Group 4 served as an uninfected control. Blood and oral fluid samples were taken every 3–4 days after HP-PRRSV challenge; on day 16 post-challenge, all pigs were euthanized, and examined pathologically.

**Results:**

The nucleotide sequence analysis of nonstructural protein 2 gene of EDRD1 and comparison with Vietnamese HP-PRRSV showed that the 39 amino acid deletion sites of EDRD1 was nearly in the same region as the 29 amino acid deletion sites of HP-PRRSV. Immunity conferred by inoculation with EDRD1 dramatically reduced viral load in the sera and tissues besides viral shedding (Group 2) compared with those in pigs infected only with HP-PRRSV (Group 3). The clinical signs and rectal temperature were significantly reduced, and the average daily weight gain was significantly improved in the EDRD1-inoculated pigs (Group 2) compared with the Group 3 pigs. Notably, no viral RNA was detected in various organs of the Group 2 pigs 16 days post-infection with HP-PRRSV, except in one pig. Therefore, the immunity induced by EDRD1 and its genetically close field isolates may play a role in reducing viremia caused by HP-PRRSV.

**Conclusions:**

The results of the present study demonstrate that pigs are highly protected against heterologous Vietnamese HP-PRRSV challenge by immunity against a Japanese local strain, EDRD1.

## Background

Porcine reproductive and respiratory syndrome (PRRS) is characterized by reproductive failure in sows and respiratory symptoms in piglets and growing pigs. The etiologic agent of PRRS is the PRRS virus (PRRSV), an enveloped, single-stranded, positive-sense RNA virus, which belongs to the newly classified genus *Rodartevirus* in the family *Arteriviridae* in the order Nidovirales [1]. PRRSV was first reported in Europe during 1990 [2] and in the United States during 1992 [3]. Two distinct genotypes of PRRSV, which share approximately 60% identity at the nucleotide level, have been described: type 1 (European genotype) and type 2 (North American genotype) [4]. Although PRRSV was generally defined as the etiologic agent of chronic infection in pigs, a recently identified highly pathogenic PRRSV (HP-PRRSV) causing high fever and mortality in animals of all ages has emerged from both types 1 and 2 [5, 6]. The type 2 HP-PRRSV isolated in China in 2006 shows a unique discontinuous 30 amino acid deletion in the nonstructural protein 2 (nsp2) gene in open reading frame (ORF) 1a [5, 7]. The virus and its evolved variants have rapidly spread throughout countries of southeast Asia, where it has caused severe economic losses [8, 9]. Countries presently free from this highly virulent PRRSV, such as Japan, remain at high risk for the invasion and spread of this virus.

In Japan, type 2 non-HP-PRRSV infection was first described in 1994 [10, 11]. Since then, although various type 2 PRRSV variants classified to some of the different lineages based on the ORF5 gene analysis has been detected all over the country [12]. Interestingly, we found that the standard strain in Japan, EDRD1, has a 39 amino acid deletion, which is in almost the same region as the nsp2 gene with the HP-PRRSV in the present study. Nsp2, a region of the ORF1 replicase protein, is highly heterogeneous and has various deletions and insertions [13]. Although the size of nsp2 is variable because of hypervariability in the central region, suggesting the existence of a nonessential region for replication in nsp2 [14], the influence of the variability of nsp2 gene to the cross-protection between PRRSVs is still unclear. However, Leng and his colleagues [15] reported that the nsp2 region of an attenuated strain of Chinese HP-PRRSV played a key role in the induction of neutralizing antibodies in piglets. Predicting the strength of protective immunity against PRRSV infections among heterogeneous viral strains is difficult based on analyzing contributing factors, such as the production of neutralizing antibodies [16] and genetic characteristics [17]. Therefore, the evaluation of the cross-immunity between PRRSVs might depend upon challenge studies of whether a vaccine can alleviate pathological conditions. To date, no reports have evaluated the protective immunity derived from infection with uniquely evolved viruses in Japan against the HP-PRRSV. The present study aimed to determine the cross-protection of a Japanese local strain, EDRD1, against heterologous type 2 HP-PRRSV challenge with a low-passage Vietnamese HP-PRRSV field isolate (strain 10,186–614) in growing pigs by evaluation of the clinical symptoms, growth parameters, viral replication, and development of antibodies against PRRSV.

## Results

### Comparative analysis of the nsp2 and ORF5 genes

**Fig. 1:**
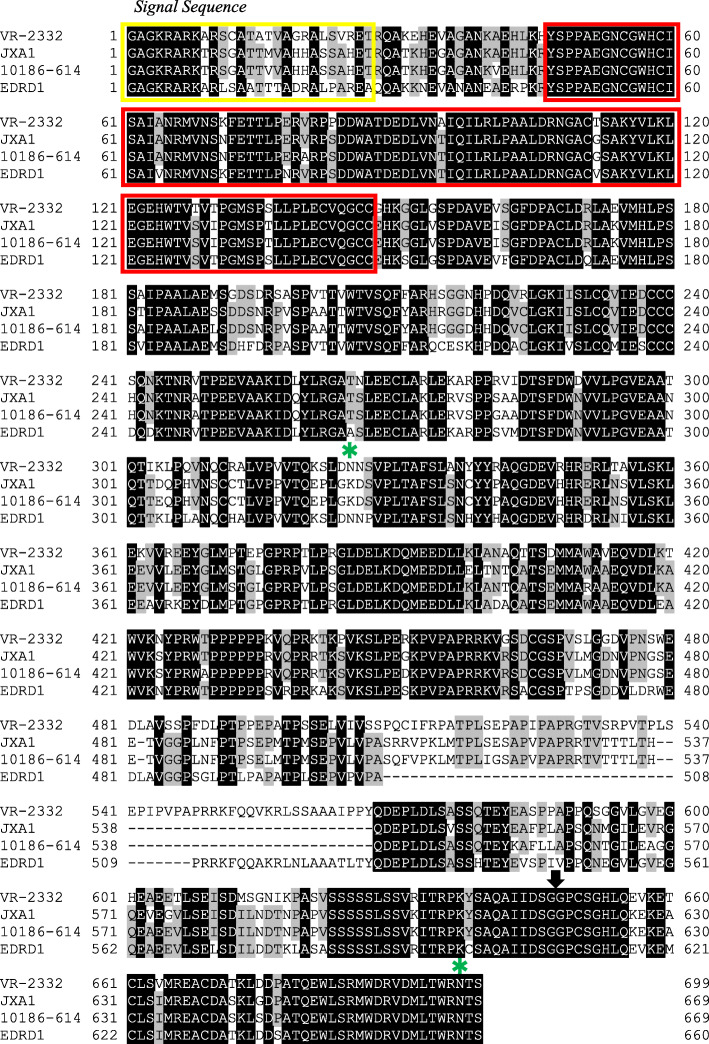
Alignment of the amino acid sequences of the nsp2 gene from divergent porcine reproductive and respiratory syndrome virus (PRRSV) strains. Genetic analysis was conducted using CLUSTAL W with Genetyx Software v14 (Genetyx, Tokyo, Japan). The black boxes indicate complete amino acid conservation (4/4), whereas gray and unshaded boxes identify less conserved residues (3/4 or less). A signal peptide was predicted using the Genetyx Software v14 (Genetyx, Tokyo, Japan) (yellow-boxed region). The completely conserved putative cysteine–protease catalytic residues are identified by the red-boxed amino acids to signify protease sequence conservation within PRRSV and equine arteritis virus. The potential *N*-glycosylation sites indicated by asterisks (*) were identified by PROSITE [18]. The conserved cysteine residue that is proposed to interact with the M protein is denoted by the downward arrow (↓)

To evaluate the similarity of the deletion site in the nsp2 gene between EDRD1 and HP-PRRSV (strain 10,186–614), we conducted comparisons of these with two other reference strains, namely, VR-2332 and JXA1. An amino acid alignment of the partial sequences of nsp2 in these four strains, in order of their pairwise identity, is shown in Fig. 1. Strain 10,186–614 contained two discontinuous deletions in the translated protein with deletion sizes of 1 and 29 amino acids corresponding with amino acid positions at 482 and 538–567, respectively, in VR-2332 nsp2. In contrast, EDRD1 contained one continuous deletion of 39 amino acids corresponding with amino acid positions 509–547 in VR-2332 nsp2. Two potential *N*-glycosylation sites were detected in VR-2332 and EDRD1 at amino acid positions 325 and 697 of VR-2332 nsp2. However, amino acid position 325 is a commonly mutated asparagine-to-lysine residue in JXA1 and 10,186–614 strains. The conserved cysteine residue proposed to interact with the matrix protein and the protease sequence that is conserved within PRRSV and equine arteritis virus were well conserved among all four strains.

ORF5 encodes glycoprotein (GP) 5, which is frequently used for the diagnostic identification of PRRSV [19]. To elucidate the genetic distances between 10,186–614 and EDRD1, we aligned GP5 among the four strains (Table 1). Alignment of PRRSV GP5 determined shared amino acid identities ranging from 87.9 to 89.5% between VR-2332 and other strains and from 88.9 to 89.9% between EDRD1 and other strains. Additionally, strains 10,186–614 and JXA1 shared 97.0% identity.

**Table 1:** Pairwise comparison of the amino acid sequences of ORF5

	% Identities to the sequences with GenBank accession no.			
Virus	U87392	EF112445	LC569874	AB288356
VR-2332	100.0	87.9	89.5	89.4
JXA1	87.9	100.0	97.0	88.9
10,186–614	89.5	97.0	100.0	89.9
EDRD1	89.4	88.9	89.9	100.0

### Clinical signs

**Fig. 2:**
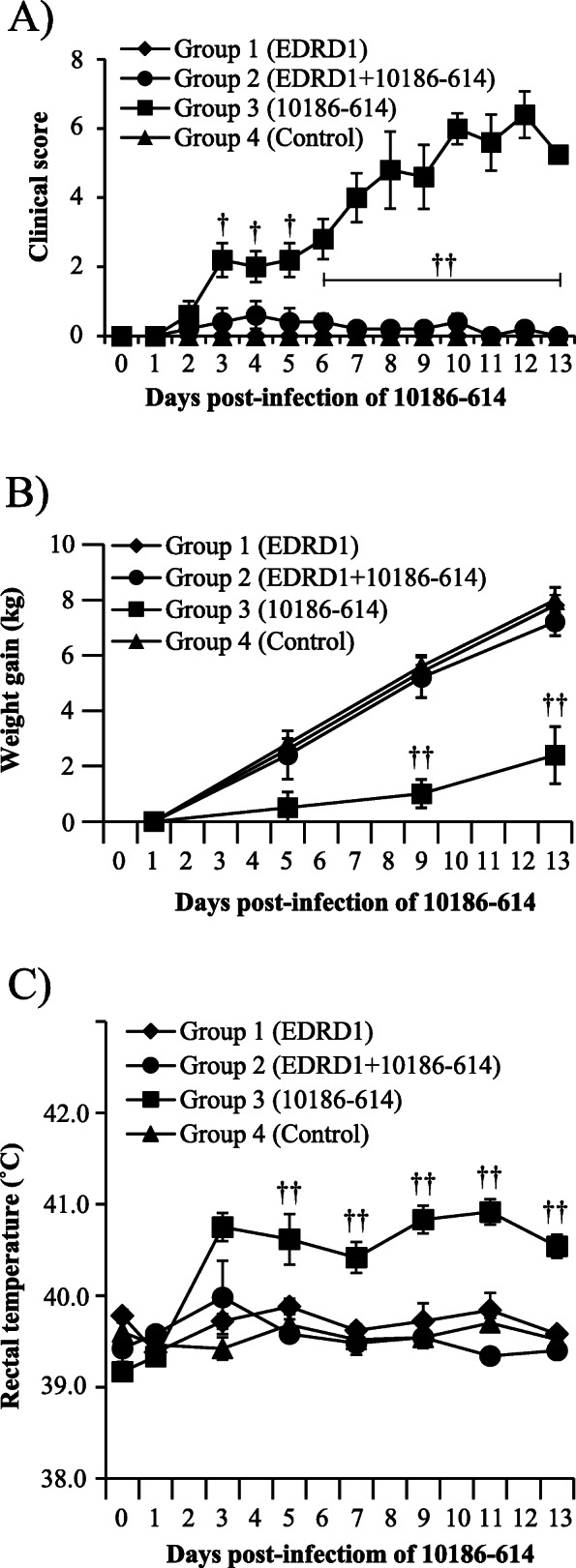
Clinical scores (**a**), weight gain (**b**), and rectal temperatures (**c**) of the pigs following challenge with strain 10,186–614. Bars indicate the standard error (SE) of the mean. Statistically significant differences between Groups 2 and 3 are indicated (^††^*P* < 0.01, ^†^0.01 < *P* < 0.05)

Clinical scores in the EDRD1-inoculated group (Group 2) were significantly lower than those in the group without EDRD1 inoculation (Group 3) from 3 days post-infection (dpi) till the end of the experiment with strain 10,186–614 (Fig. 2a). After 10,186–614 challenge, pigs in Group 3 exhibited periocular edema and reddish skin on the trunk. These pigs demonstrated a poor appetite and respiratory signs such as coughing and abdominal breathing seriously. These clinical signs persisted through the end of the experiment. Some of pigs in Group 3 lay collapsed in the recumbent position and exhibited intermittent tremor. One of these (pig G17) was euthanized on 12 dpi due to difficulty drinking water by itself. In contrast, three of five pigs in Group 2 transiently showed loss of appetite and tachypnea between 3 and 12 dpi, but the pigs recovered by 13 dpi. No clinical signs were observed in animals not challenged with strain 10,186–614 (Groups 1 and 4).

### Daily weight gain and rectal temperature

The mean daily weight gain of the pigs in Group 3 was significantly lower than that of Group 2 between 9 and 13 dpi (Fig. 2b). By the day of necropsy (16 dpi), the mean weight gain was 9.00 kg for Group 1 (EDRD1 inoculation without HP-PRRSV challenge), 10.00 kg for Group 2 (EDRD1 inoculation and HP-PRRSV challenge), 5.13 kg for Group 3 (HP-PRRSV challenge without EDRD1 inoculation), and 9.60 kg for Group 4 (uninfected controls).

The mean rectal temperature in two of five pigs (pig G9 and G12) in Group 2 increased transiently to 3 dpi, whereas that of all animals of Group 3 rapidly increased until plateauing 3 dpi (Fig. 2c). Overall, by 5 dpi and afterward, the mean rectal temperatures in Group 3 were significantly higher than those in Group 2. No significant difference arose in the mean rectal temperatures between Groups 1, 2, and 4.

### Antibody to PRRSV

**Fig. 3:**
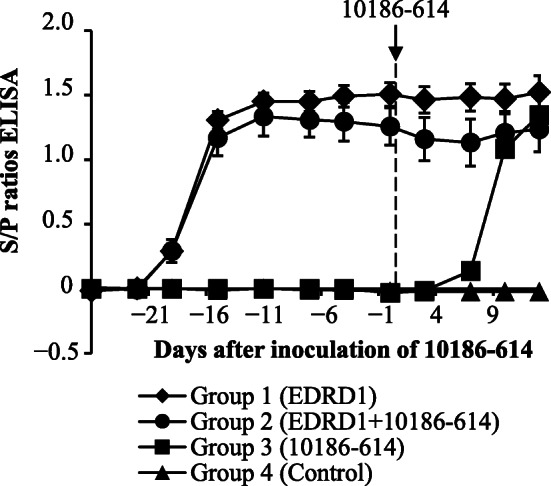
Mean titers of porcine reproductive and respiratory syndrome virus (PRRSV) antibody measured by ELISA (sample-to-positive, S/P, ratios) before and after virus challenge. Error bars indicate the standard error (SE) of the mean

Antibodies against PRRSV were observed in Groups 1 and 2 beginning at 7 days after EDRD1 inoculation in some pigs and were over the cutoff line (sample-to-positive ratio, S/P, 0.4) in all animals at 11 days after EDRD1 inoculation (− 15 dpi of the challenge) (Fig. 3). Although the S/P ratio of four of five pigs in Group 2 was 1.28, 1.24, 1.37, and 1.32, that of pig G12 was 0.63 at 11 days after EDRD1 inoculation. The S/P ratio of G12 then gradually rose very slowly compared with those of the other four pigs, and that of pig G12 was 0.92 at 0 dpi. In Group 3, the positive conversion of antibody to PRRSV was observed at 7 or 10 dpi, and the S/P ratio of viral antibody gradually rose. In contrast, no antibodies against PRRSV were observed in uninoculated Group 4.

### Viral RNA

**Fig. 4:**
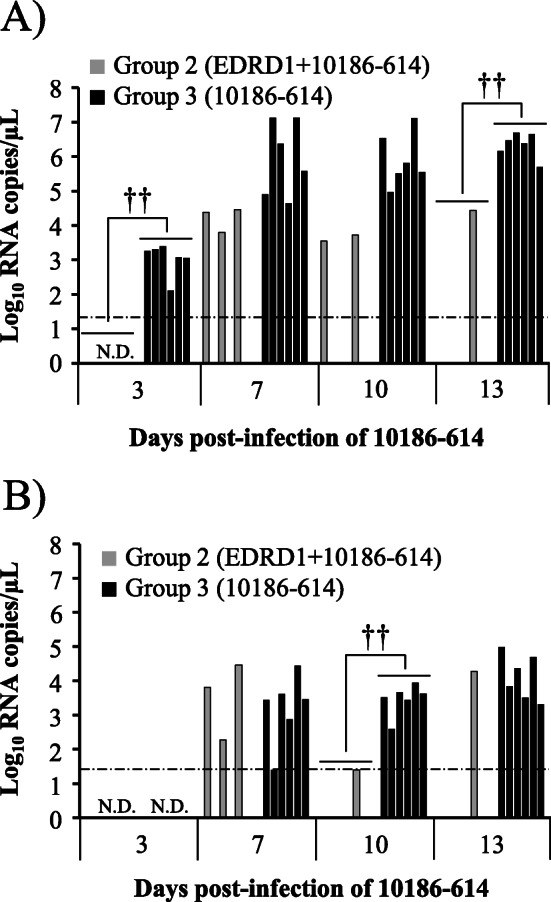
Quantity of viral RNA in the serum (**a**) and oral fluid (**b**) of the pigs following challenge with strain 10,186–614. Quantity of porcine reproductive and respiratory syndrome virus (PRRSV) RNA was measured by one-step real-time reverse transcriptase PCR. Statistically significant differences between Groups 2 and 3 are indicated (^††^*P* < 0.01). Gray squares indicate the quantity of the viral RNA of Group 2; black squares indicate those of Group 3. N.D. indicates that the viral RNA was under the detection limit of RT-qPCR. The maximum dilution value of the synthetic DNA was set as the cut off (dashed line)

**Fig. 5:**
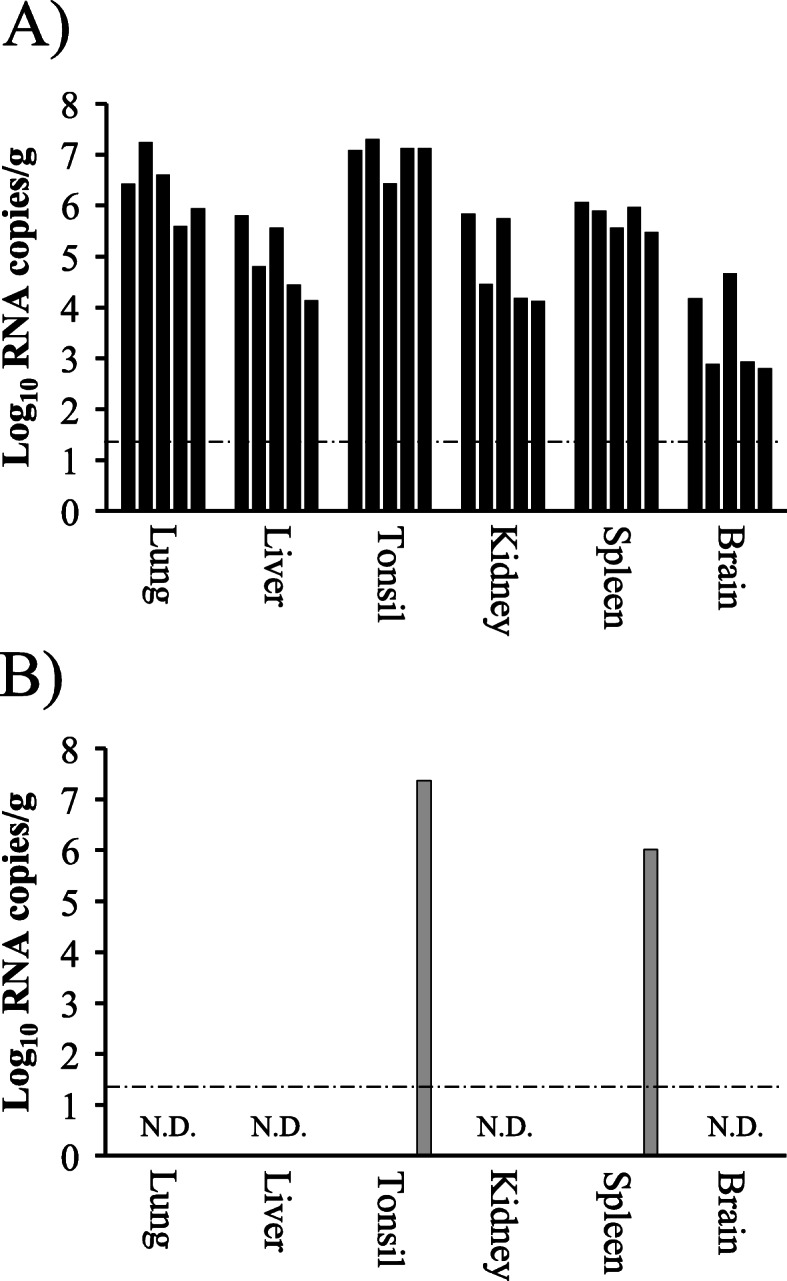
Quantity of viral RNA in each organ at 16 days post-infection (dpi) with 10,186–614. Quantity of porcine reproductive and respiratory syndrome virus (PRRSV) RNA was measured by one-step real-time reverse transcriptase PCR (RT-qPCR). Black squares indicate the quantity of the viral RNA of Group 3 (**a**); gray squares indicate those of Group 2 (**b**). N.D. indicates that the viral RNA was under the detection limit of RT-qPCR. The maximum dilution value of the synthetic DNA was set as the cut off (dashed line)

Serum viral load (viral RNA copies/mL) in Group 3 was significantly higher than that of Group 2 at 3 and 13 dpi (Fig. 4a). The mean 10,186–614 viral RNA in Group 3 pigs sharply rose at 3 and 7 dpi and plateaued afterward between 2.8 × 10^6^ and 4.9 × 10^6^ copies/mL. In contrast, the viral load in two of five pigs in Group 2 transiently rose at 7 dpi (pig G9) and 7 and 10 dpi (pig G7) and in a third pig reached 5.3 × 10^3^ to 2.9 × 10^4^ copies/mL from 7 to 13 dpi (pig G12). For the remaining two pigs in Group 2, viral RNA in both sera and oral fluid remained under the detection limit (pigs G8 and G10) after 10,186–614 challenge. Viral RNA was detected in oral fluid from all animals in Group 3 at 7, 10, and 13 dpi (Fig. 4b). The viral load in the oral fluid in Group 2 was transiently detected in pigs G7 and G9 at 7 dpi and was intermittently detected in pig G12 at 7, 10, and 13 dpi. At necropsy on 16 dpi, viral RNA was detected in all tested organs, namely, the lung, liver, tonsil, kidney, spleen, and brain of all pigs inoculated with 10,186–614 in Group 3 (Fig. 5a). Mean viral RNA copies/g tissue were approximately 5.2 × 10^6^ in the lung, 2.2 × 10^5^ in the liver, 1.3 × 10^7^ in the tonsil, 2.6 × 10^5^ in the kidney, 7.1 × 10^5^ in the spleen, and 1.3 × 10^4^ in the brain. In contrast, viral RNA was detected from only the tonsils (2.3 × 10^7^ copies/g) and spleen (1.0 × 10^6^ copies/g) of pig G12 in Group 2 (Fig. 5b). Expectedly, no 10186–614 viral RNA was found in the sera, oral fluid, or any tissues of pigs in Groups 1 and 4.

### Gross and histological lesions

**Fig. 6:**
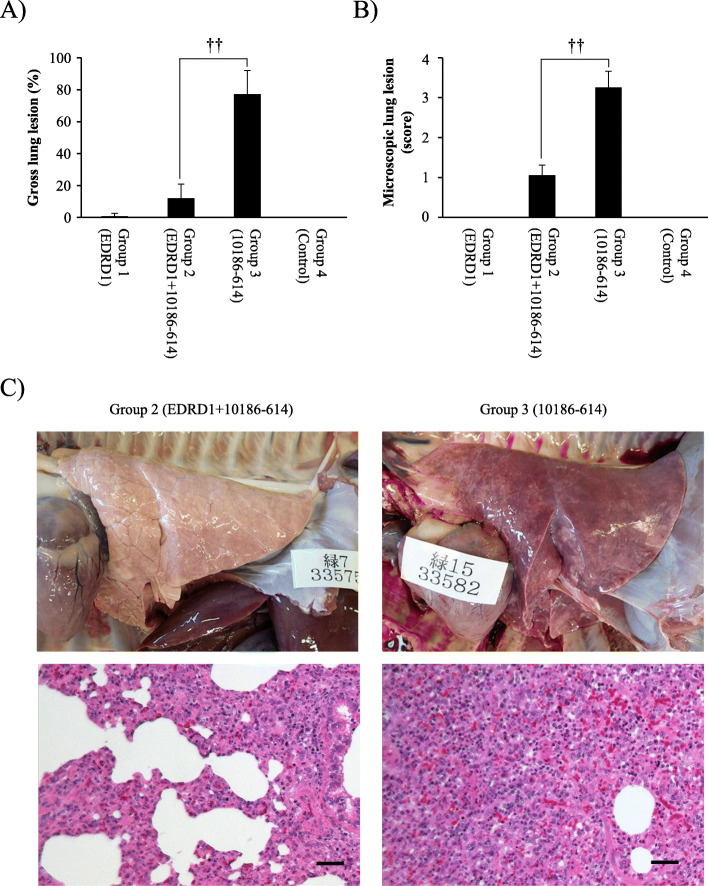
Gross and microscopic lung lesions in pigs at 16 days post-infection with 10,186–614. Gross lung lesions (%) (**a**) and microscopic lung lesions (score) (**b**) are shown in each group with mean ± standard error (SE). Statistically significant differences between Groups 2 and 3 are indicated (^††^*P* < 0.01). C: Upper panels show the left dorsal surface of the lungs; lower pictures show moderate to severe interstitial pneumonia (hematoxylin and eosin staining, bar = 200 μm) in pigs of Groups 2 and 3

Gross anatomic observation after euthanization on 16 dpi revealed prominent lesions of pneumonia and thymic atrophy in the organs of Group 3 pigs. The lung lesions in these pigs were characterized by dark-reddish pneumonia with congested edematous consolidation in the whole lobes. (Fig. 6c, upper panel). In contrast, the lungs in most pigs of Group 2 were only slightly red compared with those in Group 1. Mean (standard deviation, SD) gross lung lesion scores were 0.8% ± 1.8% in Group 1, 12.0% ± 8.9% in Group 2, 77.2% ± 14.8% in Group 3, and 0% ± 0% in Group 4, with a significant difference between Groups 2 and 3 (Fig. 6a).

In Group 3 pigs, severe interstitial pneumonia with marked accumulation of proteinaceous and cell debris in alveolar spaces were characterized histopathologically, and septal infiltration with macrophages and capillary congestion in the alveolar wall were shown (Fig. 6c, lower panel). On the other hand, slight to moderate histiolymphocytic infiltration in the alveolar wall were found in Group 2. The microscopic pneumonic score (mean ± SD) in Group 2 (1.1 ± 0.3) was significantly lower than that in Group 3 (3.3 ± 0.4) (Fig. 6b).

## Discussion

The results of the present study demonstrate that pigs inoculated with Japanese local strain EDRD1 are highly protected against a challenge infection with heterologous Vietnamese highly pathogenic type 2 PRRSV strain 10,186–614. Viral replication, viral emission in oral fluid, clinical signs, rectal temperature, and lesion severity were significantly reduced, and average daily weight gain was significantly improved in pigs immunized with EDRD1 compared with those in non-immunized animals. Therefore, the results of the present study suggest that the immunity induced by EDRD1 inoculation strongly inhibits the replication of Vietnamese HP-PRRSV in pigs.

EDRD1 historically has been used for various studies as a Japanese standard strain because its genetically closely related variants are broadly endemic in Japan [20, 21]. Yoshii and his colleagues reported that 21 of 31 Japanese field isolates contained the same deletion pattern in the nsp2 gene [22]. To evaluate details of the amino acid deletion sites, we compared the partial nsp2 gene products of EDRD1 and 10,186–614 against two historical reference strains, namely, VR-2332 and JXA1. Chinese HP-PRRSVs, including JXA1, are classified into Lineage 8/Sublineage 8.7/Subgroup 3, with 1 and 29 residues containing common deletion sites in nsp2 [23, 24]. Nucleotide sequence analysis of nsp2 of EDRD1 and comparisons among JXA1 and 10,186–614 showed that the 39 amino acid deletion site of EDRD1 (VR-2332 nsp2 amino acids 509–547) was in almost the same region as the 29 amino acid deletion site of JXA1 and 10,186–614 strains (VR-2332 nsp2 amino acids 538–567). The nsp2 ORF contains a conserved protease motif and predicted regions of the interaction with a matrix protein (Fig. 1), which are essential for the replication of PRRSV, but this ORF is highly tolerant of mutations in the large middle section. Furthermore, one of the two potential *N*-glycosylation sites (VR-2332 nsp2 amino acid 325) in that middle section was broken in the gene products of JXA1 and 10,186–614 strains.

ORF5 is one of the most variable regions of the PRRSV genome, likely because it encodes the major envelope protein and can be exposed to selective pressures [19]. To elucidate the genetic distance among the two strains that we used for the present study and the two historical reference strains, we compared their ORF5 amino acid sequences. According to the latest publication for the genetic variation of type 2 PRRSV [12], four strains were classified as follows: 10186–614 and JXA1, Lineage 8/Sublineage 8.7/Subgroup 3; VR-2332, Lineage 5/Sublineage 5.1; and EDRD1, Lineage 4. ORF5 gene alignment indicated that EDRD1 is not genetically closely related to any of the other three strains of the present study (10186–614, JXA1, or VR-2332). Therefore, the sequence analysis suggests that the high cross-immunity between EDRD1 and 10,186–614 was not caused by the level of the sequence identity, although it might be affected by changing the three-dimensional structure of Nsp2 due to nucleotide deletions. Although we did not deeply analyze the other regions of the PRRSV genomic sequences, considering that the aim of this analysis was to identify the genetic distances among the abovementioned viruses, these results could be representable of genome wide analysis.

We found that intranasal infection with 10,186–614 quickly resulted in viremia in pigs without EDRD1 inoculation (Group 3). Although we set the experiment period to 16 dpi to minimize the pain and suffering of the animals challenged with 10,186–614, one pig was prematurely euthanized because of severe symptoms. Viremia contributed to the distribution of the virus throughout every organ tested in the infected animals. The virus in Group 3 pigs was initially detected in serum samples at 3 dpi before peaking at 7 dpi and then remaining at high titers throughout the experimental period (Fig. 4a). Virus shedding at 7, 10, and 13 dpi was correlated with viremia (Fig. 4b); high quantities of viral RNA were detected in oral fluid collected from all animals. The virus may not be a part of a genetically homogenous population owing to the fact that we did not use a virus that was rescued from cDNA clone; however, these results are consistent with a previous report of viremia caused by this strain [25], indicating that our experimental design implements high experimental reproducibility. In contrast, viral RNAs were not detected at 3 dpi in the EDRD1-inoculated and challenged group and were significantly lower than those without EDRD1 inoculation at 7 and 13 dpi. We found that 10,186–614 challenge after EDRD1 inoculation significantly reduced clinical and pathological scores and fever and restored daily weight gain to normal values in pigs compared with those without EDRD1 inoculation (Fig. 2 and Fig. 6). Notably, viral RNA in both sera and oral fluid from two of five pigs in Group 2 (pigs G8 and G10) were not detectable throughout the experiment. Furthermore, viral RNA was transiently detected from sera at 7 and 10 dpi and oral fluids at 7 dpi in pig G7, and from both sera and oral fluids at 7 dpi in pig G9 in Group 2. Although the amount of viral RNA was the lowest compared with those in Group 3 pigs, only pig G12 had continued viremia at 13 dpi and excreted the virus in oral fluid. The 2.3 × 10^7^ and 1.0 × 10^6^ copies/g of viral RNA were also detected from the tonsils and spleen of pig G12. Pig G12 did not generate sufficient immunity against EDRD1 inoculation because of unknown reasons; however, no viral RNA was detected from its other organs, despite it being distributed in every organ of all challenged pigs without EDRD1 inoculation (Fig. 5). Hence, the virus distributed through the bloodstream must have been captured and processed by the immune system. To elucidate the reason for the limited protection in only pig G12 against the challenge infection, we measured the EDRD1 viral quantity in the sera using EDRD1-specific PCR primers. This pig displayed almost equal EDRD1 amounts as those of the other animals in Group 2 until the 10,186–614 challenge (data not shown). The S/P ratio quickly reached up to 1.0 by 10 dpi in all animals except pig G12; however, pig G12 reached this ratio at 36 dpi because of a prolonged antibody response. This animal seemed to be in good health because no clinical symptoms were recorded until the 10,186–614 challenge, and the daily weight gain was almost the same as those of the other pigs in Group 2. However, neither its antibodies against whole viral particles nor the serum neutralizing antibodies was sufficient to protect pig G12 from HP-PRRSV challenge. Although we recognized that further experiments using a greater number of animals will be necessary, these results indicate that those animals that are successfully immunized by EDRD1 infection are highly protected from 10,186–614 and its genetically closely related variants.

To reduce economic losses in pig production caused by Lineage 8 HP-PRRSV, some research teams have shown that presently available vaccines for non-HP-PRRSVs are partially effective in reducing symptoms and viral shedding of HP-PRRSV strains [26–28]. Similarly, we previously demonstrated that pigs immunized with the commercial type 2 PRRS MLV (modified live virus) vaccine showed significantly reduced symptoms, viremia, and virus shedding caused by 10,186–614 infection compared with those in a non-vaccinated group [25]. However, the immunity conferred by those vaccine strains could not protect the pigs from a viral infection with HP-PRRSV. In the present study, we demonstrated that pigs inoculated with EDRD1 are highly protected from 10,186–614 challenge infection. Although a herd with immunity derived from Lineage 4 field isolates might show resistance against Lineage 8 HP-PRRSV, further research is necessary to test whether the same potent immunogenicity of EDRD1 against Lineage 8 HP-PRRSV can be shared with genetically closely related strains. If the unique character of EDRD1 confers such immunity, this old standard strain may be an ideal candidate for vaccine development against this economically devastating virus in the near future.

## Conclusions

In the present study, we demonstrated that pigs are protected against acute, heterologous Vietnamese HP-PRRSV type 2 challenge by immunity against the Japanese local strain, EDRD1. Notably, two of five EDRD1-inoculated pigs showed no evidence of HP-PRRSV infection within 16 days after the challenge with this high-morbidity virus. Further research is necessary to determine whether the strong immunogenicity of EDRD1 against the economically devastating HP-PRRSV can be shared among strains that are genetically closely related.

## Methods

### Virus

HP-PRRSV (10186–614 strain) was isolated in 2010 from an infected pig with HP-PRRS in Vietnam using the monkey kidney cell line MARC-145 by three passages as described previously [29]. Dr. Tung and Dr. Inui kindly provided the isolated virus. The EDRD1 strain was cloned by three rounds of limiting dilution in swine alveolar macrophages, and the virus of the 17th passage was used for the animal experiments. Macrophages were obtained from pigs aged approximately 4 weeks old and were cultured as described previously [30]. These isolates were stored at − 80 °C until use.

### Animal experiments

Twenty-two specific pathogen free (SPF) pigs (landrace and large white crossbreds) aged 3 weeks were purchased from a closed SPF herd (Zen-Noh Livestock, Co., LTD., Tokyo, Japan), marked with an individual ear tag and randomly divided into four groups. They were negative for PRRS, pseudorabies, porcine epidemic diarrhea, transmissible gastroenteritis, atrophic rhinitis, *Mycoplasma pneumonia*, swine dysentery, salmonellosis, toxoplasma, and actinobacillosis. Pigs were also confirmed negative for antibodies to PRRSV before the experiment using a commercially available enzyme-linked immunosorbent assay (ELISA) kit (PRRS X3 Ab Test; IDEXX Laboratories, Westbrook, ME, USA). Group 1 (*N* = 5; pigs G1–G5) and Group 2 (*N* = 6; pigs G7–G12. G11 was excluded from subsequent experiments at 12 days after EDRD1 inoculation due to the accidental death.) were inoculated with 1 mL nasal spray containing strain EDRD1 at a median tissue culture infectious dose (TCID_50_) of 1 × 10^5^. After 26 days, Groups 2 and 3 were challenged with 1 mL nasal spray containing 1 × 10^5^ TCID_50_ of strain 10,186–614 (N = 6; pigs G13–G18). The remaining five pigs were nasally administered 1 mL medium on the same schedule as the other groups and were used as uninfected controls (Group 4; pigs G19–G23). The sample size (N = 6) was determined as the 4 animals for the minimum number of the statistical analysis and an additional 2 animals because of a predicted 10–20% mortality rate according to the previous report [25]. The pigs of each group were housed in a separate room of the animal facility where they received a commercial diet and were monitored for rectal temperature, clinical signs, and body weight throughout the experimental period. The rooms were cleaned daily by qualified personnel. All pigs were euthanized and necropsied at 16 dpi with 10,186–614 for pathologic and virologic assays except pig G17, which was euthanized at 12 dpi at a humane endpoint to release the animal from pain and suffering. At the end of euthanasia, the piglets were anaesthetized intramuscularly with a mixture of 20 mg/kg ketamine hydrochloride (Ketalar; Daiichi Sankyo Propharma Co., Tokyo, Japan) and 0.3 mg/kg xylazine (Selactal; Bayer Medical Co., Wuppertal, Germany) prior to euthanization by the intravenous administration of a barbiturate (Somnopentyl, Kyoritsu Seiyaku, Tokyo, Japan) according to the manufacturer’s instruction. Then the piglets were humanely bled to death. All the data set collected from live animals were used for subsequent analysis. To monitor the viral RNA, serum separated from 1 mL of whole blood was collected at − 26, − 22, − 19, − 15, − 11, − 7, − 4, 0, 3, 7, 10, and 13 dpi, and oral fluids were collected at 0, 3, 7, 10, and 13 dpi. Approximately 1–2 mL of oral fluid samples collected using Salivette Cotton (Sarstedt, Nümbrecht, Germany), were immediately centrifuged, and the supernatant was stored at − 80 °C until use. Blood and oral fluid were collected from the animals in a random order each time. Animal experiments were performed according to regulations and guidelines of the Animal Ethics Committee of the National Institute of Animal Health (approval number 13–047). To conduct the acts of randomization and blinding appropriately, blood sampling was conducted by various researchers barring the 1st and 2nd authors; viral and pathological examination were performed separately by the 1st and 2nd authors.

### RT-qPCR

During necropsy, lung, liver, tonsil, kidney, spleen, and brain tissues were collected for quantification of viral RNA. Viral RNA from 0.2 mL of serum and oral fluid was extracted using a QIAamp Viral RNA Mini kit (Qiagen, Tokyo, Japan); tissue viral RNA was extracted using a Qiagen RNeasy Mini kit (Qiagen). Viral RNA extracts were then used as templates for one-step reverse transcriptase quantitative-polymerase chain reaction (RT-qPCR) with a One-Step SYBR® PrimeScript™ RT-PCR kit II (Perfect Real-Time; Takara Bio Inc., Shiga, Japan). RNA extraction and RT-qPCR assay were conducted according to the manufacturer’s instructions. To detect the two types of viral RNAs separately, two pairs of PCR primers were designed based on the nsp2 gene of 10186–614 (forward, 5′-AACTGTGACAACAACGC-3′, and reverse, 5′-CGATGATGGCTTGAGCTGAGT-3′) and EDRD1 (forward, 5′ -ATCCCTGTGCCCGCACC-3′, and reverse, 5′-GTCGATAATGGCTTGAGCTGAAC-3′). For the standard curve, positive control DNA was generated for the synthetic gene 10186–614 (partial, 267 bp) and EDRD1 nsp2 (partial, 317 bp) synthesized from forward to reverse primers by GeneArt® Strings DNA Fragments (Thermo Fisher Scientific, Waltham, MA, USA). A linear standard curve was generated for each RT-qPCR reaction using serial dilutions of each synthetic DNA fragment. The C_T_ values are valid only between the minimum and maximum values obtained using the standard RNA. Fluorescence data were analyzed using PE 7500 Sequence Detection System Software Version 1 (Thermo Fisher Scientific).

### Pathological examinations

The scoring system used for visible pneumonia and histopathologic pneumonic lesions followed a previous paper [31] with modification to match the characteristics of lesions from an experimental infection with HP-PRRSV. A visual examination and microscopic examination were conducted according to the methods presented in our previous study (24). No significant pathogens were isolated from the tissues collected from pigs in any of the groups at necropsy.

### Statistical analysis

Body temperature and viral RNA values were analyzed using an unpaired *t*-test. Microscopic pneumonia scores were analyzed using the Mann–Whitney *U-*test with the “EZR” package in the R statistical environment (Foundation for Statistical Computing). A *P* value of < 0.05 was considered statistically significant.

## Data Availability

The datasets used and/or analyzed during the present study are available from the corresponding author on reasonable request.

## Abbreviations

DNA: Deoxyribonucleic acid
dpi: Days post-infection
ELISA: Enzyme-linked immunosorbent assay
GP: Glycoprotein
HP-PRRSV: Highly pathogenic porcine reproductive and respiratory syndrome virus
MLV: Modified live virus
N.D.: Not detected
Nsp: Nonstructural protein
ORF: Open reading frame
PRRS: Porcine reproductive and respiratory syndrome
PRRSV: Porcine reproductive and respiratory syndrome virus
RT-qPCR: Reverse transcriptase-quantitative polymerase chain reaction
RNA: Ribonucleic acid
SAM: Swine alveolar macrophage
SD: Standard deviation
SE: Standard error
S/P ratio: Sample-to-positive ratio
SPF: Specific pathogen free
TCID_50_: Median tissue culture infectious dose
